# Hypoxia inducible factor signaling and experimental persistent pulmonary hypertension of the newborn

**DOI:** 10.3389/fphar.2015.00047

**Published:** 2015-03-11

**Authors:** Stephen Wedgwood, Satyan Lakshminrusimha, Paul T. Schumacker, Robin H. Steinhorn

**Affiliations:** ^1^Department of Pediatrics, University of California Davis Medical CenterSacramento, CA, USA; ^2^Department of Pediatrics, State University of New York at BuffaloBuffalo, NY, USA; ^3^Department of Pediatrics, Northwestern UniversityChicago, IL, USA

**Keywords:** pulmonary hypertension, hypoxia, vascular stress, reactive oxygen species, HIF-1α

## Abstract

**Background**: Mitochondrial reactive oxygen species (ROS) levels and nuclear factor kappa-light-chain-enhancer of activated B cells (NFκB) activity are increased in a lamb model of persistent pulmonary hypertension of the newborn (PPHN). These events can trigger hypoxia inducible factor (HIF) signaling in response to hypoxia, which has been shown to contribute to pulmonary vascular remodeling in rodent models of pulmonary hypertension. However, the role of HIF signaling in chronic intrauterine pulmonary hypertension is not well understood.

**Aim**: To determine if HIF signaling is increased in the lamb model of PPHN, and to identify the underlying mechanisms.

**Results**: PPHN was induced in lambs by antenatal ligation of the ductus arteriosus at 128 days gestation. After 9 days, lungs and pulmonary artery smooth muscle cells (PASMC) were isolated from control and PPHN lambs. HIF-1α expression was increased in PPHN lungs and HIF activity was increased in PPHN PASMC relative to controls. Hypoxia increased HIF activity to a greater degree in PPHN vs. control PASMC. Control PASMC were exposed to cyclic stretch at 1 Hz and 15% elongation for 24 h, as an *in vitro* model of vascular stress. Stretch increased HIF activity, which was attenuated by inhibition of mitochondrial complex III and NFκB.

**Conclusion**: Increased HIF signaling in PPHN is triggered by stretch, via mechanisms involving mitochondrial ROS and NFκB. Hypoxia substantially amplifies HIF activity in PPHN vascular cells. Targeting these signaling molecules may attenuate and reverse pulmonary vascular remodeling associated with PPHN.

## Introduction

At birth, the lung replaces the placenta as the organ of gas exchange. When the newborn takes its first breath, the sudden increase in lung oxygenation reverses hypoxic pulmonary vasoconstriction, resulting in an 8–10 fold increase in pulmonary blood flow (Dawes et al., 1953). Complex physiological and biochemical processes facilitate the fetal to newborn transition, and abnormal lung development and/or vascular dysfunction may disrupt these events. Failure to adapt to postnatal life results in persistent pulmonary hypertension of the newborn (PPHN), characterized by elevated pulmonary vascular resistance, right-to-left extrapulmonary shunting of deoxygenated blood and severe hypoxemia (Steinhorn, 2010). Some degree of PPHN complicates the course of approximately 10% of term and preterm infants with respiratory failure, and increases the risk of death, pulmonary morbidity, and neurodevelopmental impairment (Konduri et al., 2004). PPHN is associated with a severely remodeled pulmonary vasculature, including increased thickness of the smooth muscle layer within small pulmonary arteries and abnormal extension of this muscle to non-muscular arteries (Haworth and Reid, 1976). The extent of pulmonary vascular remodeling correlates with the severity of the disease, although the *in utero* mechanisms that disrupt normal development and adaptation of the pulmonary circulation remain poorly understood.

A better understanding of the underlying mechanisms that trigger pulmonary vascular remodeling and vasoconstriction is warranted to develop new therapies and preventative strategies for PPHN. In fetal lambs, constriction of the ductus arteriosus produces fetal and neonatal pulmonary hypertension (Abman et al., 1989; Morin, 1989; Wild et al., 1989; Black et al., 1998). After delivery, these lambs have persistent hypoxemia and elevation of PVR, providing an important experimental model of human infants with PPHN. This model of PPHN is associated with striking pulmonary vascular remodeling along with elevated levels of reactive oxygen species (ROS) in the lungs and pulmonary arteries (Brennan et al., 2003; Wedgwood et al., 2005), and ROS have been implicated in impaired pulmonary vasodilation and increased smooth muscle proliferation.

After birth, chronic hypoxia also induces pulmonary vascular abnormalities characteristic of PPHN in several animal models including newborn piglets (Allen and Haworth, 1986; Tulloh et al., 1997) and newborn mice (Ambalavanan et al., 2005). Hypoxia inducible factors (HIF) are highly conserved transcription factors that are expressed in multiple cell types, and control the oxygen-dependent expression of numerous genes (Shimoda and Laurie, 1985). Because heterozygous mice lacking one HIF-1α allele display attenuated hypoxia-induced pulmonary vascular remodeling and pulmonary hypertension (Yu et al., 1999), HIFs are believed to play an important role in pulmonary vascular signaling.

*In vitro* studies have identified ROS as intermediates in hypoxic stabilization of HIF-1α protein (Guzy et al., 2005), suggesting that elevated ROS levels and subsequent HIF-induced transcription may contribute to vasoconstriction and vascular remodeling in PPHN. However, the involvement of HIFs in the development of pulmonary hypertension and smooth muscle proliferation in PPHN lambs is currently unknown. The purpose of this study is to quantify HIF-1α expression in lungs and pulmonary artery smooth muscle cells (PASMC) isolated from PPHN lambs relative to controls, and to use *in vitro* techniques to investigate the mechanisms involved. Identification of a major regulator of abnormal gene expression may reveal novel therapeutic targets for the prevention and treatment of PPHN.

## Materials and methods

### Animals

This protocol was approved by the Laboratory Animal Care committee at University at Buffalo and University of California, Davis. Time-dated pregnant ewes were obtained from Swartz family farm in Attica, NY. Fetal lambs underwent antenatal ligation of the ductus arteriosus at 128 days gestation (term 143–145 days) to induce pulmonary hypertension as previously described (Morin, 1989; Zayek et al., 1993). Lambs were delivered 9 days later and sacrificed with an overdose of thiopental sodium and exsanguination before their first breath. Fifth generation pulmonary arteries and lung tissue were collected for further analysis.

### Western blot analysis

Lung tissue was homogenized and total protein collected using the PARIS kit (Ambion, Austin, TX) as previously described (Farrow et al., 2008a). Protein lysates from PASMC were prepared using 1X Mg-lysis buffer (Upstate, Charlottesville, VA) supplemented with a protease inhibitor cocktail (Sigma). Protein concentration was measured using the Bradford method (Bradford, 1976). Total protein (40 μg) was separated on a 4–20% SDS-polyacrylamide gel (Biorad, Hercules, CA) and then transferred to a nitrocellulose membrane (Amersham, Arlington Heights, IL). Western blot was then performed as previously described (Farrow et al., 2008a,b). Briefly, membranes were blocked at room temperature with 5% non-fat dry milk in Tris-buffered saline containing 0.1% Tween 20 (1X TBST) and were then incubated overnight at 4°C with a mouse anti-HIF-1α antibody (Novus Biologicals, Littleton, CO) in 5% milk + 1X TBST at a 1:1000 dilution. The membranes were washed and incubated with an anti-mouse secondary antibody conjugated to horseradish peroxidase (Pierce, Rockford, IL) diluted 1:1000 in 5% milk + 1X TBST. Membranes were washed and exposed via chemiluminescence (Pierce). Bands were analyzed using a Digital Science Image Station (Kodak, Rochester, NY). Expression within each Western blot was normalized to β-actin.

### Immunocytochemistry

Cells were seeded onto microscope slides and fixed with 4% paraformaldehyde. Slides were blocked with 5% BSA (Sigma) + 1X TBST at room temperature for 1 h and then probed overnight at 4°C with anti-HIF-1α antibody (1:100 dilution). Slides were washed and probed with a Rhodamine Red Goat anti-mouse secondary antibody (Molecular Probes/Invitrogen) at a 1:200 dilution in 5% BSA. Localization and expression was visualized with a Nikon Eclipse TE-300 fluorescent microscope with excitation at 518 nm and emission at 605 nm. Fluorescent images were captured using a CoolSnap digital camera with Metamorph imaging software (Molecular Devices, Sunnyvale, CA).

### Cell culture

Primary cultures of pulmonary arterial smooth muscle cells (PASMC) from control and PPHN fetal lambs were isolated by the explant technique and maintained in culture as described previously (Wedgwood et al., 2001). Briefly, a segment of the main pulmonary artery from 136-day-old fetal lambs was excised and placed in a sterile 100 mm dish containing Dulbecco's modified Eagle's medium (DMEM) supplemented with 1 g/l glucose (Mediatech, Herndon, VA). The segment was stripped of adventitia with a sterile forceps. The main pulmonary artery segment was then cut longitudinally to open the vessel, and the endothelial layer was removed by gentle rubbing with a cell scraper. The vessel was then cut into 2-mm segments, inverted, and placed on a collagen-coated 35-mm tissue culture dish. A drop of DMEM containing 10% fetal bovine serum (FBS; HyClone, Logan, UT), antibiotics (MediaTech), and antimycotics (Mediatech) was then added, and the cells were grown overnight at 37°C in a humidified atmosphere with 5% CO_2_–95% air. The next day, an additional 2 ml of complete medium were added. The growth medium was subsequently changed every 2 days. When SMC islands could be observed under the microscope, the tissue segment was removed and the individual cell islands were subcloned. Identity was confirmed as PASMCs by immunostaining (>99% positive) with antibodies against α-smooth muscle actin, calponin and caldesmon. This was taken as evidence that cultures were not contaminated with fibroblasts or with endothelial cells. All cultures for subsequent experiments were maintained in DMEM supplemented with 10% fetal calf serum (Hyclone), antibiotics (MediaTech), and antimycotics (MediaTech) at 37°C in a humidified atmosphere with 5% CO_2_–95% air. Cells were used between passages 2–5.

### Plasmid DNA transfection and luciferase assays

HIF promoter activity was determined using the pGL4.42 [luc2P/HRE/Hygro] plasmid containing 4 consensus HRE sequences fused to a luciferase reporter (HRE-luc) and NFκB promoter activity was determined using the pGL4.32 [*luc2P*/NF-κB-RE/Hygro] plasmid containing five consensus NFκB response elements fused to a luciferase reporter (NFκB-luc) (both Promega, Madison, WI). PASMC were co-transfected with 4 μg of plasmid DNA and 0.1 μg pRL-CMV Vector (Promega) on a 10 cm^2^ tissue culture plate at 60% confluence, using Lipofectamine (Gibco BRL) according to the manufacturer's instructions. After 24 h cells were split onto 6-well plates (hypoxia) or BioFlex plates (cyclic stretch) and allowed to adhere. Cells were synchronized in serum-free DMEM for 24 h, returned to DMEM containing 10% serum and exposed to hypoxia or to cyclic stretch as described below. Luciferase activity in protein extracts was determined using the Dual-Luciferase Reporter Assay System (Promega) and a Femtomaster FB12 luminometer (Zylux). Activity was normalized to the internal renilla luciferase control to correct for differences in transfection efficiencies. Where appropriate, cells were treated with 1 μM myxothiazol or 10 μM Helenalin (Calbiochem) prior to luciferase assays.

### Hypoxia

Cells were transfected with the HRE-luc promoter construct plasmid as described above. PASMCs were returned to DMEM containing 10% serum and maintained in an incubator with 21% O_2_–5% CO_2_ or exposed to 5% O_2_–5% CO_2_in a Coy chamber (CoyLabs, Grass Lake, MI) for 24 h.

### Cyclic stretch

Cells were transfected with HRE-luc and NFκB-luc promoter construct plasmids as described above, seeded onto six-well BioFlex plates coated with collagen type IV (FlexCell) and subjected to biaxial cyclic stretch using the FlexCell 3000 Strain Unit. Plates were placed on a loading station and stretched by applying an oscillatory vacuum to the underside of the membranes. Cells were stretched at a frequency of 1 Hz with 15% amplitude for 24 h in accordance with a previous study (Quinn et al., 2002).

### Statistical analysis

Means ± SEM were calculated from individual experiments and expressed as fold change relative to the same internal control: control lungs (HIF-1α expression), control PASMC in normoxia (hypoxia experiments) or to static control cells treated with vehicle where appropriate (stretch experiments). Results were analyzed by two-sided unpaired *t*-test or by ANOVA with Newman-Keuls *post-hoc* testing using Prism software (GraphPad Software Inc., San Diego, CA). Statistical significance was set at *p* < 0.05.

## Results

**Figure 1:**
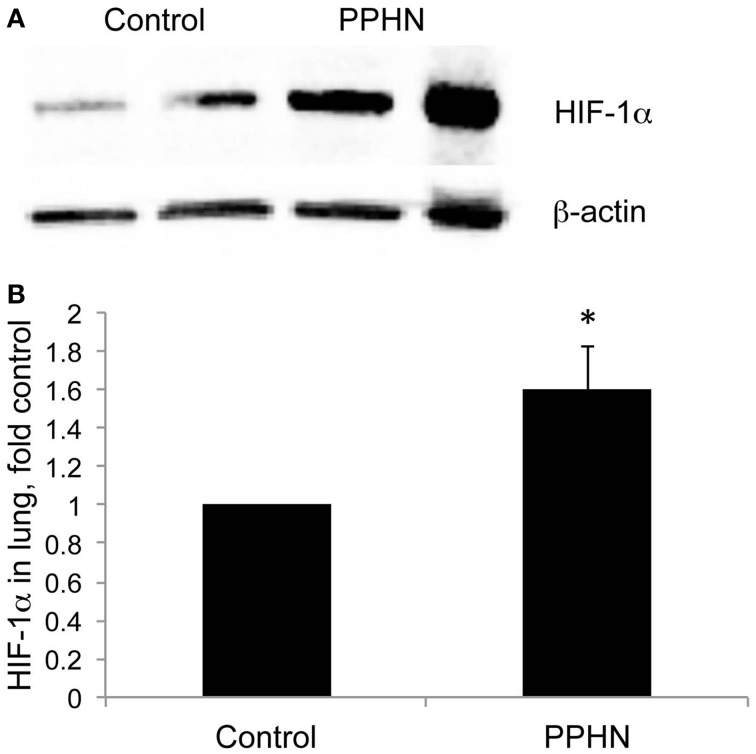
**HIF-1α expression is increased in PPHN lungs (A) Representative blots for HIF-1α from control and PPHN lungs**. Lysates from two different animals in each group are depicted. **(B)** Expression levels were normalized to β-actin levels and expressed as fold change relative to controls. ^*^p < 0.05. *N* = 4 animals.

Total lung HIF-1α protein levels were increased in lung samples from PPHN lambs relative to controls as detected by Western blotting (Figure 1A). When normalized to β-actin, normoxic expression of HIF-1α was 1.6-fold higher in PPHN lungs (Figure 1B, ^*^p < 0.05).

**Figure 2:**
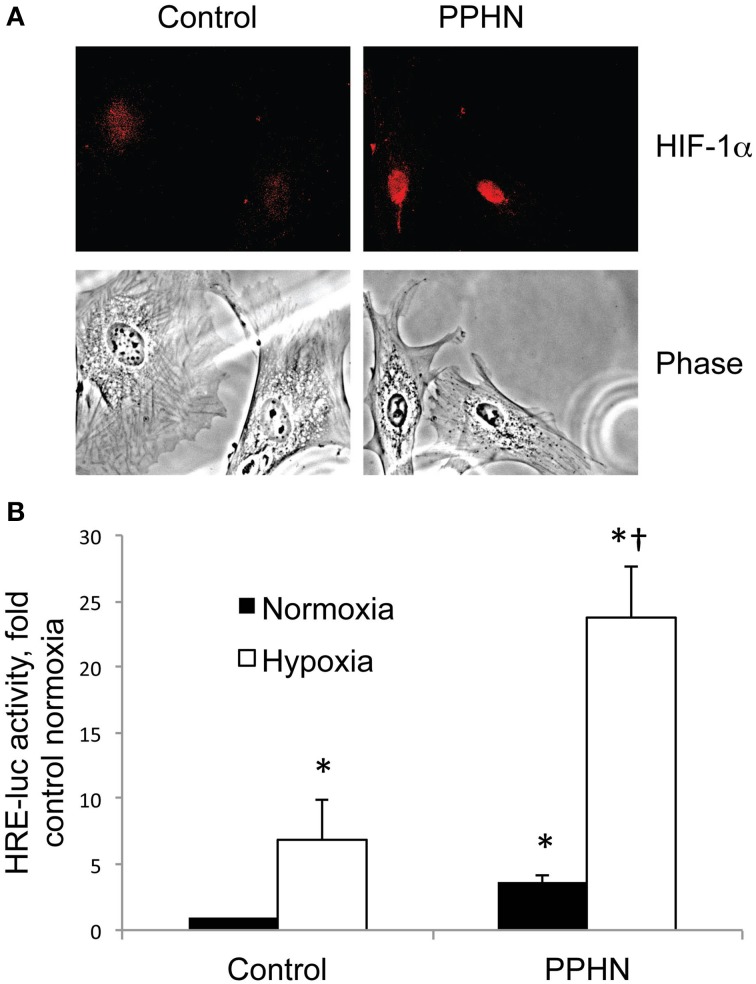
**HIF-1α nuclear expression and activity is increased in PPHN PASMC (A) Representative immunocytochemistry images from control and PPHN PASMC depicting nuclear localization of HIF-1α and the corresponding light phase image. (B)** Control and PPHN PASMC were transfected with a plasmid containing four consensus HRE sequences upstream of a luciferase reporter (HRE-luc). Cells were maintained in normoxia or exposed to 1.5% O_2_ for 24 h before assay. Relative light units were determined in a luminometer, normalized to an internal renilla luciferase control plasmid and expressed as fold change relative to control cell lysates. ^*^p < 0.05 vs. control normoxia; ^†^*p* < 0.05 vs. control hypoxia and PPHN normoxia. *N* = 4.

Nuclear HIF-1α protein levels were higher in PPHN vs. control PASMC cultured in normoxia as detected by immunocytochemistry (Figure 2A). To quantify cellular HIF activity we utilized a plasmid containing four consensus hypoxia response elements (HRE), a DNA binding sequence for HIFs in target gene promoters, fused to a luciferase reporter (HRE-luc). Normoxic HIF activity was 3.6-fold higher in PPHN PASMC relative to controls (Figure 2B, ^*^p < 0.05). Hypoxia dramatically increased HIF activity to 6.9-fold and 23.8-fold in control and PPHN PASMC respectively relative to normoxic control cells (Figure 2B, ^*^p < 0.05). Under hypoxic conditions, HIF activity was 3.4-fold higher in PPHN PASMC relative to control cells and 6.6-fold higher in PPHN PASMC relative to normoxic PPHN PASMC (Figure 2B, ^†^*p* < 0.05).

**Figure 3:**
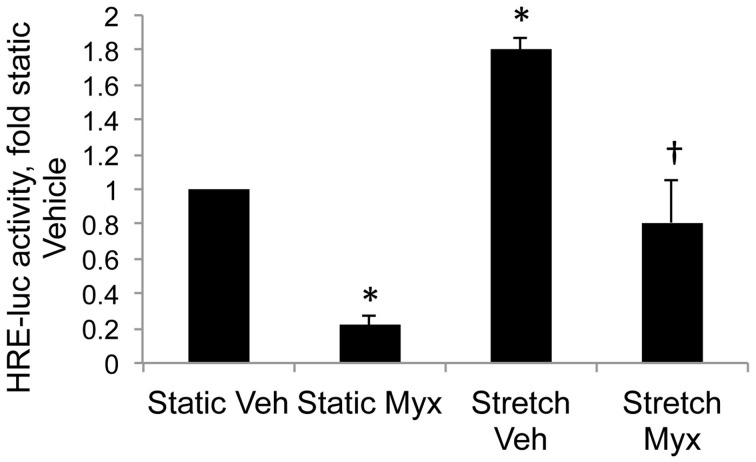
**Inhibition of mitochondrial complex III attenuates stretch-induced HIF activity**. Control PASMC were transfected with the HRE-luc plasmid, treated with vehicle (Veh) or with 1 μM myxothiazol (myx) to inhibit complex III, and subjected to 24 h cyclic stretch at 1 Hz and 15% elongation. Relative light units were determined in a luminometer, normalized to an internal renilla luciferase control plasmid and expressed as fold change relative to control cell lysates. ^*^p < 0.05 vs. static vehicle; ^†^*p* < 0.05 vs. stretch vehicle. *N* ≥ 4.

Cyclic stretch increased HIF activity in control PASMC by 1.8-fold relative to static cells (Figure 3, ^*^p < 0.05). Inhibition of mitochondrial complex III with myxothiazol decreased basal HIF activity in static cells (Figure 3, ^*^p < 0.05) Stretch-induced HIF activity was attenuated by the mitochondrial complex III inhibitor myxothiazol (Figure 3, ^†^*p* < 0.05).

**Figure 4:**
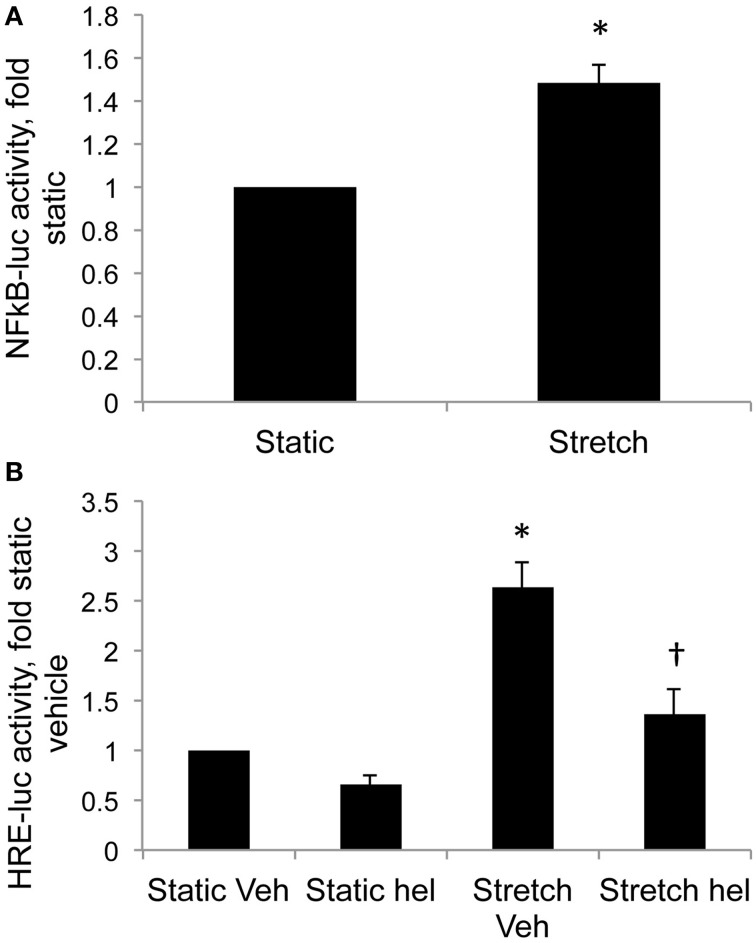
**Stretch increases NFκB activity and NFκB inhibition attenuates stretch-induced HIF activity. (A)** Control PASMC were transfected with a plasmid containing five consensus NFκB promoter elements fused to luciferase reporter (NFκB-luc) and subjected to 24 h cyclic stretch at 1 Hz and 15% elongation. **(B)** Control PASMC were transfected with the HRE-luc plasmid, treated with vehicle (Veh) or with 10 μM helenalin (hel) to inhibit NFκB, and subjected to 24 h cyclic stretch at 1 Hz and 15% elongation. Relative light units were determined in a luminometer, normalized to an internal renilla luciferase control plasmid and expressed relative to control cell lysates. ^*^p < 0.05 vs. static **(A)** or static vehicle **(B)**. ^†^*p* < 0.05 vs. stretch vehicle. *N* = 4.

Cyclic stretch increased NFκB promoter activity in control PASMC by 1.5-fold relative to static cells (Figure 4A, *p* < 0.05). Stretch-induced HIF activity in control PASMC was attenuated by the NFκB inhibitor helenalin (Figure 4B, *p* < 0.05).

## Discussion

**Figure 5:**
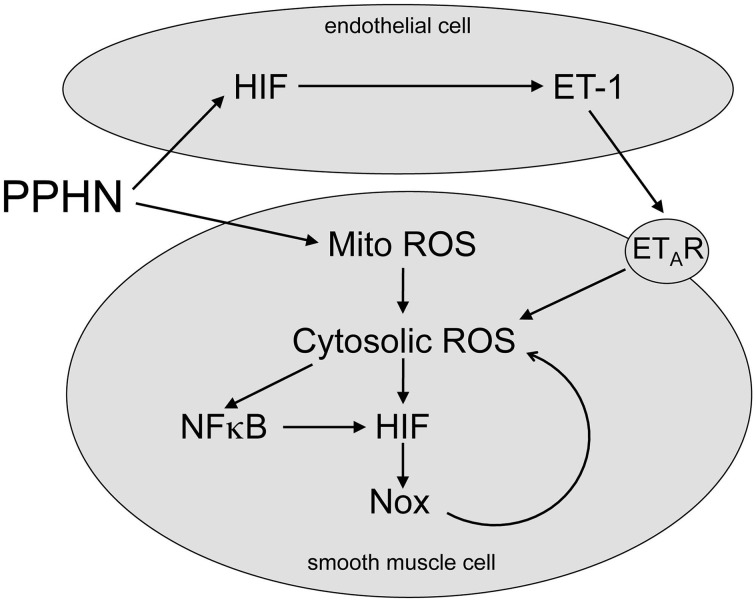
**Proposed HIF signaling pathway in PPHN**. PPHN increases mitochondrial ROS (Mito ROS) in smooth muscle cells, resulting in an increase in cytosolic ROS. This increases NFκB and HIF activities, leading to increased expression of target genes including NADPH oxidases (Nox). This stimulates a feed forward mechanism that gives a sustained increase in Cyto ROS. PPHN also increases HIF activity in endothelial cells, leading to an increase in expression and release of ET-1. ET-1 activates ET_A_ receptors (ET_A_R) on adjacent smooth muscle cells, leading to vasoconstriction and increased cytosolic ROS. Cytosolic ROS can stimulate vascular remodeling and vasoconstriction as discussed in the text.

Newborns with PPHN that die shortly after birth display extreme hypertensive structural remodeling (Geggel and Reid, 1984), suggesting that the most severe cases of disease stem from chronic intrauterine stress. Determining the intrauterine events that alter pulmonary vascular reactivity and structure is essential to improve early detection and treatment. In this study we identified elevated HIF-1α expression in the lungs (Figure 1), and elevated HIF activity in PASMC (Figure 2), as potential regulators of abnormal gene expression in PPHN lambs. Antenatal surgical closure of the ductus arteriosus results in a sustained elevation of pulmonary arterial pressure in PPHN lambs (Storme et al., 1999). Cyclic stretch mimics increased pulmonary artery pressure, and in the current study we found that cyclic stretch increased HIF activity in PASMC isolated from control lambs via mechanisms involving mitochondrial complex III (Figure 3) and NFκB (Figure 4). Targeting the signaling pathways that stimulate HIF activity and the genes that are upregulated by HIFs (Figure 5) may represent a novel strategy for the treatment of PPHN (Table 1).

**Table 1:** **Proposed targets, roles in PPHN and potential pharmacologic approaches**.

**Target**	**Proposed role in PPHN**	**Potential pharmacologic approaches**
Complex III	Generates ROS in response to stretch, hypoxia	Terpestacin, MitoQ
NFκB	Amplifies gene expression in response to ROS, stretch, hypoxia, inflammation	Pyrrolidine dithiocarbamate, glucocorticoids
HIF	Amplifies gene expression in response to ROS, stretch, hypoxia	Glyceollins
Nox	Generate sustained ROS in response to stretch, hypoxia Dysregulate enzymes important for NO signaling	Pyrazolopyridine derivatives (GKT137831), triazolo pyrimidine derivatives (VAS 2870)
ET_A_R	Vasoconstriction and vascular smooth muscle growth	Bosentan, Ambrisentan, Macitentin

Elevated HIF expression and activity in lungs and PASMC may contribute to abnormal gene expression that has been demonstrated previously in PPHN lambs. HIFs are heterodimers consisting of oxygen-sensitive α-subunits (HIF-1α, HIF-2α) and constitutively expressed β subunits. Hypoxia stabilizes the α subunit leading to nuclear accumulation and activation of multiple target genes (Shimoda and Laurie, 1985). The fetus is programmed to develop under hypoxic conditions, and HIF expression is essential to normal fetal lung development. Basal HIF expression has been reported to be higher in the fetal relative to the adult lung (Resnik et al., 2007), and deletion of the HIF gene is lethal during fetal life. Tamoxifen-induced silencing of smooth muscle HIF-1α expression attenuates pulmonary vascular remodeling and pulmonary hypertension in chronically hypoxic mice (Ball et al., 2014), suggesting a central role for HIF-1α in the pulmonary vascular response to hypoxia. Interestingly, increased lung HIF-1α expression is also evident in a lamb model of pulmonary hypertension secondary to congenital heart disease and vascular stress from increased pulmonary blood flow (Diebold et al., 2010a). Together these data suggest that elevated HIF signaling may contribute to pulmonary vascular remodeling and pulmonary hypertension in PPHN.

We found that hypoxia increased HIF activity in PASMC by 6.9-fold in control PASMC and 6.6-fold in PPHN PASMC (Figure 2B). Since normoxic HIF activity is almost 4-fold higher in PPHN PASMC (Figure 2B), the hypoxic intrauterine environment of the developing fetus may exacerbate abnormal HIF signaling during disease progression. Previous reports suggest that smooth muscle HIF-1α may contribute to the sustained vasoconstrictor response to hypoxia (Ball et al., 2014), and our findings may partly explain why PPHN lambs have greatly enhanced vasoconstrictor responses in response to hypoxia (Lakshminrusimha et al., 2009). However, the mechanisms that elevate HIF activity under normoxic conditions are poorly understood.

*In vitro* models can be useful to investigate novel signaling pathways. In PPHN lambs, occlusion of the ductus arteriosus generates a myogenic response where stretch-induced vasoconstriction opposes the increase in pulmonary blood flow, resulting in a sustained elevation of pulmonary arterial pressure (Storme et al., 1999). In the current study we exposed control PASMC to cyclic stretch to mimic increased pulmonary artery pressure, and demonstrated that 24 h stretch increased HIF activity relative to static controls (Figure 3). Stretch also increases expression of HIF-1α in rat aortic SMC (Chang et al., 2003) in agreement with our results. We also found that stretch-induced HIF activity was attenuated by the mitochondrial complex III inhibitor myxothiazol (Figure 3). Hypoxic stabilization of HIF-1α involves the release of ROS from mitochondrial complex III into the cytosol (Guzy et al., 2005). Under normoxic conditions, PASMC isolated from PPHN lambs have increased levels of mitochondrial (Farrow et al., 2010) and cytosolic (Wedgwood et al., 2011) ROS relative to controls. We recently reported that exposure of control PASMC to cyclic stretch increases cytosolic ROS levels (Shah et al., 2013), while mitochondrial inhibitors attenuate stretch-induced ROS in pulmonary artery endothelial cells (Ali et al., 2006). Together these data suggest that elevated mitochondrial and cytosolic ROS levels may contribute to increased HIF-1α expression and activity in PPHN lungs and PASMC, and that these abnormalities are sustained independent of hypoxia. Targeting the pathways that stabilize HIFs under normoxic conditions may attenuate abnormal gene expression characteristic of PPHN, and provide therapeutic strategies that can reverse its pulmonary vascular remodeling.

In the current study we extend our findings by showing that stretch increased nuclear factor kappa B (NFκB) activity, and that stretch-induced HIF activity was attenuated by the NFκB inhibitor helenalin (Figure 4). NFκB upregulates HIF-1α transcription in response to hypoxia in PASMC (Belaiba et al., 2007), and we previously found elevated NFκB activity in PPHN PASMC relative to controls (Wedgwood et al., 2013). From these data we speculate that cyclic stretch triggers a release of ROS from the mitochondria into the cytosol, thereby activating NFκB and HIF and stimulating the expression of their target genes. We also speculate that similar mechanisms are active in PPHN PASMC (Figure 5).

These pathways present a number of possible therapeutic targets (Figure 5 and Table 1). Attenuating ROS production from mitochondrial complex III and/or inhibiting its target, NFκB, may prove effective in decreasing abnormal HIF-mediated gene expression in PPHN. Mitochondrial complex III inhibitors including terpestacin and MitoQ are under investigation and have been used in mice to inhibit tumor angiogenesis (Jung et al., 2010). In addition, pharmacological inhibition (Sawada et al., 2007) and nanoparticle delivery of an NFκB decoy (Kimura et al., 2009) attenuate pulmonary hypertension in rats, and our previous findings suggest that the beneficial effects of glucocorticoids could be partly mediated through NFκB inhibition (Perez et al., 2014).

While direct inhibition of HIF could prevent or reverse the vascular changes of pulmonary hypertension, currently only a few of the putative small molecule inhibitors of HIF are progressing through preclinical trials as anti-cancer agents (Galie et al., 2004). Recently glyceollins, a group of novel phytoalexins isolated from soybean, were found to decrease microvessel density in solid tumor tissues via mechanisms involving decreased HIF-1α protein synthesis (Lee et al., 2015). Furthermore, the effects of HIF inhibition would need to be balanced with the requirement of HIF for normal fetal lung development.

The proteins encoded by HIF-regulated genes may also represent attractive targets to attenuate the effects of abnormal HIF signaling in PPHN. NADPH oxidases (Nox) are a major source of vascular ROS and contribute to multiple cardiovascular diseases (Lassegue and Griendling, 2009). mRNA and protein levels of Nox2 (Wedgwood et al., 2012) and Nox4 (Wedgwood et al., 2013) are elevated in PPHN lungs and PASMC, and these Nox subunits are regulated by HIF-1α (Diebold et al., 2010b). This raises the intriguing possibility of a feed forward mechanism in PPHN, whereby Nox-derived ROS activate HIF, leading to increased Nox expression and ROS generation. Another target for HIF is the potent vasoconstrictor ET-1 (Galie et al., 2004), and ET-1 mRNA levels are elevated in PPHN lambs (Black et al., 1998). ET-1 stimulates proliferation via increased ROS (Wedgwood et al., 2001) and increases HIF-1α expression (Pisarcik et al., 2013) in PASMC. These data provide further evidence of a feed forward mechanism in PPHN involving ROS, Nox, HIFs and ET-1. ET-1 stimulates vasoconstriction mainly via ET_A_ receptors, and selective ET_A_ receptor antagonists have proved to be successful in several animal models of neonatal pulmonary hypertension (Kuo, 2001; Perreault et al., 2001; Wagenaar et al., 2013).

In summary, our data suggest that cyclic stretch induces a PPHN phenotype in control PASMC, including elevated ROS, HIF and NFκB signaling, and may represent a useful *in vitro* tool to study the abnormal *in utero* events that trigger PPHN. However, there are several limitations to the study. Ideally, control PASMC would be exposed to stretch and hypoxia simultaneously to mimic the *in utero* PPHN environment. Also, co-culture experiments would expose PASMC to vasoactive molecules including ET-1 that are released by endothelial cells in response to stretch. In this study we did not monitor HIF activity *in vivo* and the lack of available small molecule inhibitors of HIFs means that we are currently unable to determine the effects of attenuated HIF signaling on pulmonary hypertension and vascular remodeling in PPHN lambs. However, this study has identified components of the HIF signaling pathway that may contribute to the pathogenesis of PPHN, and further studies are warranted *in vivo* and *in vitro* to investigate further the mechanisms involved. These studies will improve our current detection and treatment strategies for babies with PPHN.

### Conflict of interest statement

The authors declare that the research was conducted in the absence of any commercial or financial relationships that could be construed as a potential conflict of interest.
